# i-Genome: A database to summarize oligonucleotide data in genomes

**DOI:** 10.1186/1471-2164-5-78

**Published:** 2004-10-09

**Authors:** Feng-Mao Lin, Hsien-Da Huang, Yu-Chung Chang, Jorng-Tzong Horng

**Affiliations:** 1 Department of Computer Science and Information Engineering National Central University, Chung-Li 320, Taiwan; 2 Department of Biological Science and Technology, Institute of Bioinformatics National Chiao Tung University, Hsin-Chu 300, Taiwan; 3 Department of Biotechnology, Ming Chuan University, Taipei 111, Taiwan; 4 Department of Life Science, National Central University, Chung-Li 320, Taiwan

**Keywords:** repeat, genome index, oligonucleotide, database

## Abstract

**Background:**

Information on the occurrence of sequence features in genomes is crucial to comparative genomics, evolutionary analysis, the analyses of regulatory sequences and the quantitative evaluation of sequences. Computing the frequencies and the occurrences of a pattern in complete genomes is time-consuming.

**Results:**

The proposed database provides information about sequence features generated by exhaustively computing the sequences of the complete genome. The repetitive elements in the eukaryotic genomes, such as LINEs, SINEs, Alu and LTR, are obtained from Repbase. The database supports various complete genomes including human, yeast, worm, and 128 microbial genomes.

**Conclusions:**

This investigation presents and implements an efficiently computational approach to accumulate the occurrences of the oligonucleotides or patterns in complete genomes. A database is established to maintain the information of the sequence features, including the distributions of oligonucleotide, the gene distribution, the distribution of repetitive elements in genomes and the occurrences of the oligonucleotides. The database can provide more effective and efficient way to access the repetitive features in genomes.

## Background

During the last decade, many genomes have been successfully and completely sequenced. Summarized information about the oligonucleotides in genomes provides biologists, who interests in the evolution and growth of genomes, to work in comparative genomics, oligonucleotide probe design, primer design and the analyses of genomic repetitive features.

The computation of the occurrences and the frequency of all oligonucleotides in a complete genome is very elaborate and time-consuming, especially when the genome size is very large, such as the human and mouse genomes. A database that summarizes the occurrences and the frequencies of oligonucleotides in complete genomes can facilitate the biological and the statistical analyses of genomes.

The contents of the database can be used in many biological applications, such as comparative genomics and evolution analyses [1,2], the prediction of regulatory sequences by detecting the over-represented oligonucleotides [3-8] and primer/probe design based on the uniqueness of oligonucleotides [9]. Table 1 shows the biological applications of the database entries. The entries in the database are divided into two types, namely, the occurrence positions of oligonucleotides and the frequencies of oligonucleotides. The oligonucleotide occurrences and the oligonucleotide frequencies in both the coding regions and the non-coding regions are summarized. For instance, these information can be used in statistical analyses to study the over-representation of the regulatory sequences in upstream promoter regions in genes. van Helden *et al. *systematically searched the promoter regions of potentially co-regulated genes for over-represented oligonucleotides which may be transcription factor binding sites [3]. They presented a simple and fast method for isolating DNA binding sites for transcription factors from families of co-regulated genes, illustrating their results using *Saccharomyces cerevisiae. *Although conceptually simple, the algorithm efficiently extracted the upstream regulatory sequences that had been previously been determined experimentally for most of the yeast regulatory families already analyzed. Other studies [4-8,10-12] on the prediction of gene regulatory sequences have been based on oligonucleotide analysis.

**Table 1:** Applications and the relevant data in the database.

**Database entries**	**Entry types**	**Biological applications**
Oligonucleotide occurrences	Positions	1. Oligonucleotide analysis for regulatory sequences 2. Oligonucleotide probe design 3. Primer design
Oligonucleotide frequencies	Counts	1. Oligonucleotide analysis for regulatory sequences 2. Evolutionary analysis 3. Oligonucleotide probe design 4. Primer design
Gene coding regions	Positions	1. Oligonucleotide analysis for regulatory sequences 2. Evolutionary analysis 3. Oligonucleotide probe design 4. Primer design
Repetitive element frequencies (LINE, SINE, Alu, and so on)	Counts	Evolutionary analysis
Repetitive element occurrences	Positions	Evolutionary analysis
Tandem repeats	Positions	Prediction for genetic disease marker

Hsieh *et al. *[1] investigated the oligonucleotide distributions of typical microbial genomes and found that the microbial genomes have the statistical characteristics of a much shorter DNA sequence. This peculiar property supports an evolutionary model in which a genome evolves by random mutation but grows primarily by random segmental duplication.

Repetitive elements, including LINEs, SINEs, LTR and Alu, can be investigated in evolution analysis [2]. It has been estimated that at least 43% of the human genome is occupied by four major classes of interspersed repetitive elements – LINEs, SINEs, LTR elements and DNA transposons [2]. Their analysis has yielded some insights into the evolution of the human genome. The tandem repeats provided in our database can be used for forensic analysis and the study of genetic diseases [13-16].

Another application of the established database is to facilitate the design of primers to amplify specific regions of the genomic sequence. The basic concept is that the sequences of primers from 15 to 40 bps should be unique, unlike the repetitive oligonucleotides in our database. Additionally, our database maintains the repetitive oligonucleotides that facilitate the design of oligonucleotide probes to allow the selection of signature oligonucleotides when identifying different organisms using DNA arrays [9]. The user can query oligonucleotides whose lengths exceed a threshold, such as 15 bps, to determine whether the oligonucleotides are repetitive. The non-repetitive regions of the target sequences without repetitive oligonucleotides can be used as the signatures for the target genomes.

## Construction and content

### Data sources and contents

The proposed database provides information about sequence features generated by exhaustively computing the sequences of the complete genome. The data sources including the complete genomes and the gene annotation information are obtained from GenBank [17]. The repetitive elements in the eukaryotic genomes, such as LINEs, SINEs, Alu and LTR, are obtained from Repbase [18]. The database supports a range of complete genomes including human, yeast, worm, and 128 microbial genomes. The Appendix lists the organisms supported in the database [see additional file 1]. 

The occurrences and the frequencies of oligonucleotides from one to 50 base pairs are generated and accumulated from each of the complete genome sequence. Inputting the sequence of the oligonucleotide returns the positions of the oligonucleotides. Additionally, both the occurrences and the frequencies of the repetitive elements such as LINEs, SINEs, Alu and LTR are provided by computationally scanning whole genome sequences. The tandem repeats are computationally detected by the tandem repeat finder [19]. The database also provides the gene annotation information. For instance, Table 2 presents the number of occurrences of repetitive oligonucleotides in yeast. The oligonucleotide "ACCCTA" occurs 2,724 times in the yeast genome, 822 times upstream of a gene (-600 ~-1 bp, +1 bp denotes the gene translational start postion) and 793 times in the coding regions. The counts of the occurrence of ecah oligonucleotide between one and 50 bps are present in the database.

**Table 2:** Number of occurrences of the repetitive oligonucleotides in yeast genome

**Repetitive oligonucleotide**	**Amount of occurrences**			**Repetitive oligonucleotide**	**Amount of occurrences**		
	**Genome**	**Up-streams**	**Coding region**		**Genome**	**Up-streams**	**Coding region**
ACCCTA	2,724	822	793	CAATCCA	1,895	655	343
ACCCTC	2,917	881	795	CGTCTCC	592	199	148
AGTACT	3,073	933	879	CGTCTGA	652	196	165
AGTAGA	6,673	1,970	1,798	ACAAACTA	594	179	183
AGTAGC	4,912	1,545	1,299	ACAAACTC	514	175	112
GATACC	4,829	1,638	1,005	CACAGAAAC	146	38	46
GATAGA	7,030	2,163	1,807	CACAGAAGA	164	57	39
TGGTAA	10,513	3,493	2,214	ACATATAAAAA	54	9	29
TGTAAA	11,364	3,439	3,418	ACATATAAAAC	139	34	56
AAGGGGA	1,172	299	421	ACATATAAAAG	36	7	22
AAGGGGC	626	142	256	ACTTATGTCATC	57	17	23
AGAGTGG	983	310	271	ACTTCTAGTATA	159	44	67
AGAGTTA	1,859	610	441	ACTTTTTTTTCT	32	5	21
CAATCAG	1,358	445	320	ACTTTTTTTTTC	50	6	33

### Data Generation

A software is implemented to index systematically a complete genome sequence into a suffix-array using a perfect match approach [20]. This index is only able to find the perfect match for any oligonucleotide. The user can thus use it to find the positions of a designated oligonucleotide in a genome sequence. For each genome, the occurrences of all oligonucleotides shorter than 50 bps can be efficiently searched for. The occurrence is the position of the oligonucleotide in genome. The frequency is the count of oligonucleotide occurrences in a region. The regions are the complete genome, the coding regions and the non-coding regions. Frequencies of oligonucleotides with different lengths are stored in different flat-files. For example, the two chromosomes of the *Vibrio cholerae *genome are processed separately to allow the computation of the occurrences of oligonucleotides in each chromosomal sequence. RepeatMasker [21] and the repetitive element database, Repbase [18], are used to search the instances of the repetitive elements in eukaryotic genomes. The tandem repeat finder (TRF) is used to find the tandem repeats in genomes [19]. The TRF and RepeatMasker can find the instances of repetitive elements with imperfect matches. The settings used here for each software is described in below.

The Tandem Repeat Finder uses seven parameters. These are match score, mismatch score, indel score, probability of match and insertion, minimum score of alignment and the maximum of tandem repeat pattern size. The corresponding values used here are 2, 7, 7, 80, 10, 20 and 500. The values are the default suggestions found in Tandem Repeat Finder documentation.

The transposable elements (TEs) are detected by RepeatMasker. TEs in each genome are identified using the complete dataset available from REPbase Updates [Please add the citation]. The sensitivity and the speed of RepeatMasker are set as the default values.

### Utility

Table 3 presents the two output formats – flat-files and the web query interface with a filtering function. In the flat-file format, the fields of each oligonucleotide (in a chromosome) include the sequences, the number of occurrences in the chromosome, the number of occurrences in the coding regions and the number of occurrences in the non-coding regions. The user that requires a large amount of such data can download them in this format [1].

**Table 3:** Output styles of the database.

**Database entries**	**Entry types**	**Output formats**
Oligonucleotide occurrences	Positions	Web interface
Oligonucleotide frequencies	Counts	Web interface and flat-file
Gene coding regions	Positions	Web interface
Repetitive element frequencies (LINE, SINE, Alu, and so on)	Counts	Web interface and flat-file
Repetitive element occurrences	Positions	Web interface
Tandem repeats	Positions	Web interface

**Figure 1:**
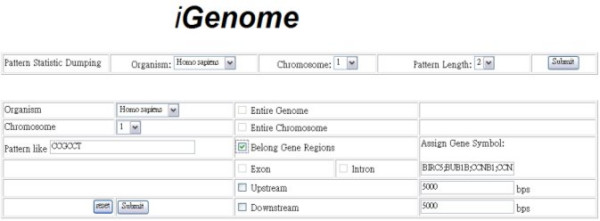
Web query interface (1/2).

**Figure 2:**
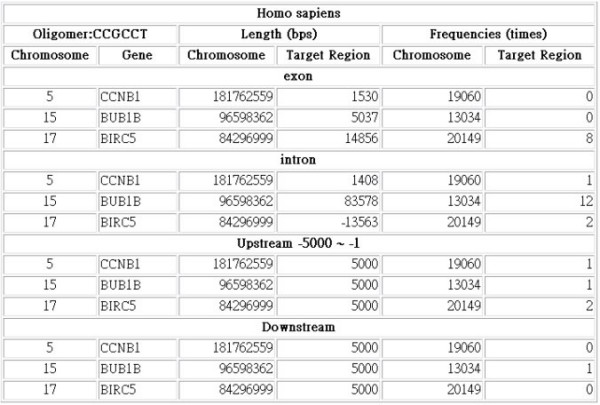
Web query interface (2/2).

**Figure 3:**
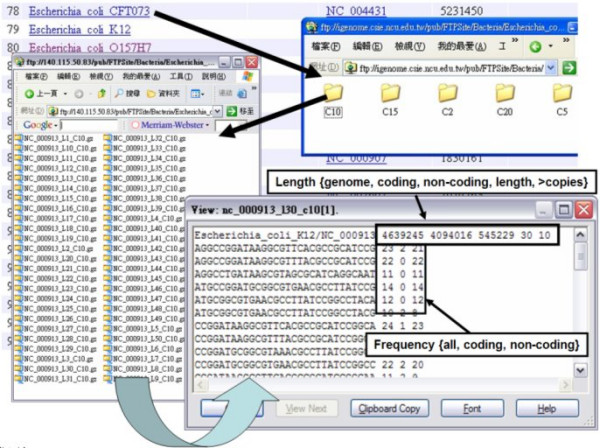
Database entries in flat-file format.

The web interface enables users to query the occurrences of an oligonucleotide in a genome and the number of occurrences in each chromosome. Figure 1 shows this approach. The occurrences of the repetitive elements and the tandem repeats in the established database can also be queried via the web interface, as in the example given in Fig 2. Figure 3 depicts the flat-file format of oligonucleotide frequencies. The first row in the flat-file presents the basic information for the oligonucleotide frequencies and the fields are the chromosome sequence/NCBI accession number, the length of the chromosome sequence, the size of coding regions, the size of non-coding regions, the length of the oligonucleotides and the minimum number of copies of the oligonucleotide. The directories labeled C10 are the files that contain the counts of oligonucleotides with at least ten occurrences in genome. Each file name includes the sequence/NCBI accession number, the length of oligonucleotides and the minimum occurrences of oligonucleotides. For example, "NC 000913 L30 C10" is the oligonucleotides, which are 30 nucleotides in length and have at least 10 occurrences in the genome.

**Figure 4:**
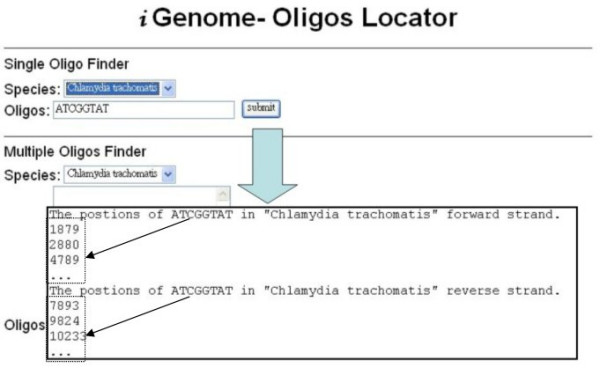
The occurrence positions of the oligonucleotide are found by Oligos Locator.

Figure 4 shows the web interface for the occurrences of a specific oligonucleotide. The user submits the query oligonucleotide and selects particular species; the positions of the oligonucleotide are then shown. The first line is the user submitted data. Following this information are the positions of oligonucleotides in forward strand. This is followed by the same information for the reverse strand.

## Conclusions

We have constructed the databases of both the oligonucleotide occurrence locations and their frequencies in the coding and the non-coding regions in complete genomes. The data in flat-file format can be downloaded directly for further analyses in several biological applications. The user may also use the web interface to query and access the database contents. The database also provides a filtering function for retrieving the information about oligonucleotides under search conditions specified by the users. Furthermore, the database provides the occurrences and the frequencies of other repetitive elements, such as LINE, SINE, Alu and tandem repeats in genomes.

## Availability and requirements

The database is now available at 

## Authors' contributions

FML implements the software and refinements the system. HDH conceives of the study and drafted the manuscripts. YCC and JTH participates the design and coordination. All authors read and approved the final manuscripts.

## Supplementary Material

Additional File 1 Appendix listing the organisms supported in the database. Click here for file
