# Supplementary material for: i-Genome: A database to summarize oligonucleotide data in genomes
Source: BMC Genomics. 2004 Oct 9;5:78. doi: 10.1186/1471-2164-5-78 (PMC526275; doi:10.1186/1471-2164-5-78)
Supplement: Additional File 1 — Appendix listing the organisms supported in the database. [file 1471-2164-5-78-S1.doc]

**Appendix**

| **Organisms** |
| --- |
| *Arabidopsis thaliana* |
| *Saccharomyces cerevisiae* (Yeast) |
| *Caenorhabditis elegans* (Worm) |
| *Homo sapiens* (Human) |
| *Aeropyrum pernix* |
| *Agrobacterium tumefaciens* C58 Cereon |
| *Agrobacterium tumefaciens* C58 UWash |
| *Aquifex aeolicus* |
| *Archaeoglobus fulgidus* |
| *Bacillus anthracis* Ames |
| *Bacillus cereus* ATCC14579 |
| *Bacillus halodurans* |
| *Bacillus subtilis* |
| *Bacteroides thetaiotaomicron* VPI-5482 |
| *Bifidobacterium longum* |
| *Blochmannia floridanus* |
| *Borrelia burgdorferi* |
| *Bradyrhizobium japonicum* |
| *Brucella melitensis* |
| *Brucella suis* 1330 |
| *Buchnera aphidicola* |
| *Buchnera aphidicola* Sg |
| *Buchnera* sp. |
| *Campylobacter jejuni* |
| *Caulobacter crescentus* |
| *Chlamydia muridarum* |
| *Chlamydia trachomatis* |
| *Chlamydophila caviae* |
| *Chlamydophila pneumoniae* AR39 |
| *Chlamydophila pneumoniae* CWL029 |
| *Chlamydophila pneumoniae* J138 |
| *Chlamydophila pneumoniae* TW 183 |
| *Chlorobium tepidum* TLS |
| *Clostridium acetobutylicum* |
| *Clostridium perfringens* |
| *Clostridium tetani* E88 |
| *Corynebacterium efficiens* YS-314 |
| *Corynebacterium glutamicum* |
| *Coxiella burnetii* |
| *Deinococcus radiodurans* |
| *Enterococcus faecalis* V583 |
| *Escherichia coli* CFT073 |
| *Escherichia coli* K12 |
| *Escherichia coli* O157H7 |
| *Escherichia coli* O157H7 EDL933 |
| *Fusobacterium nucleatum* |
| *Haemophilus ducreyi* 35000HP |
| *Haemophilus influenzae* |
| *Halobacterium* sp. |
| *Helicobacter hepaticus* |
| *Helicobacter pylori* 26695 |
| *Helicobacter pylori* J99 |
| *Lactobacillus plantarum* |
| *Lactococcus lactis* |
| *Leptospira interrogans* |
| *Listeria innocua* |
| *Listeria monocytogenes* |
| *Mesorhizobium loti* |
| *Methanobacterium thermoautotrophicum* |
| *Methanococcus jannaschii* |
| *Methanopyrus kandleri* |
| *Methanosarcina acetivorans* |
| *Methanosarcina mazei* |
| *Mycobacterium leprae* |
| *Mycobacterium tuberculosis* CDC1551 |
| *Mycobacterium tuberculosis* H37Rv |
| *Mycoplasma gallisepticum* |
| *Mycoplasma genitalium* |
| *Mycoplasma penetrans* |
| *Mycoplasma pneumoniae* |
| *Mycoplasma pulmonis* |
| *Neisseria meningitidis* MC58 |
| *Neisseria meningitidis* Z2491 |
| *Nitrosomonas europaea* |
| *Nostoc* sp. |
| *Oceanobacillus iheyensis* |
| *Pasteurella multocida* |
| *Pirellula* sp. |
| *Prochlorococcus marinus* |
| *Pseudomonas aeruginosa* |
| *Pseudomonas putida* KT2440 |
| *Pseudomonas syringae* |
| *Pyrobaculum aerophilum* |
| *Pyrococcus abyssi* |
| *Pyrococcus furiosus* |
| *Pyrococcus horikoshii* |
| *Ralstonia solanacearum* |
| *Rickettsia conorii* |
| *Rickettsia prowazekii* |
| *Salmonella typhi* |
| *Salmonella typhimurium* LT2 |
| *Salmonella typhi* Ty2 |
| *Shewanella oneidensis* |
| *Shigella flexneri* 2a |
| *Shigella flexneri* 2a 2457T |
| *Sinorhizobium meliloti* |
| *Staphylococcus aureus* Mu50 |
| *Staphylococcus aureus* MW2 |
| *Staphylococcus aureus* N315 |
| *Staphylococcus epidermidis* ATCC 12228 |
| *Streptococcus agalactiae* 2603 |
| *Streptococcus agalactiae* NEM316 |
| *Streptococcus mutans* |
| *Streptococcus pneumoniae* R6 |
| *Streptococcus pneumoniae* TIGR4 |
| *Streptococcus pyogenes* |
| *Streptococcus pyogenes* MGAS315 |
| *Streptococcus pyogenes* MGAS8232 |
| *Streptococcus pyogenes* SSI-1 |
| *Streptomyces avermitilis* |
| *Streptomyces coelicolor* |
| *Sulfolobus solfataricus* |
| *Sulfolobus tokodaii* |
| *Synechocystis* PCC6803 |
| *Thermoanaerobacter tengcongensis* |
| *Thermoplasma acidophilum* |
| *Thermoplasma volcanium* |
| *Thermosynechococcus elongatus* |
| *Thermotoga maritima* |
| *Treponema pallidum* |
| *Tropheryma whipplei* TW08 27 |
| *Tropheryma whipplei* Twist |
| *Ureaplasma urealyticum* |
| *Vibrio cholerae* |
| *Vibrio parahaemolyticus* |
| *Vibrio vulnificus* CMCP6 |
| *Wigglesworthia brevipalpis* |
| *Xanthomonas campestris* |
| *Xanthomonas citri* |
| *Xylella fastidiosa* |
| *Xylella fastidiosa* Temecula1 |
| *Yersinia pestis* CO92 |
| *Yersinia pestis* KIM |
